# Distribution and diversity of aquatic macroinvertebrate assemblages in a semi-arid region earmarked for shale gas exploration (Eastern Cape Karoo, South Africa)

**DOI:** 10.1371/journal.pone.0178559

**Published:** 2017-06-02

**Authors:** Annah Mabidi, Matthew S. Bird, Renzo Perissinotto

**Affiliations:** 1 DST/NRF Research Chair in Shallow Water Ecosystems, Nelson Mandela Metropolitan University, Port Elizabeth, South Africa; 2 Africa Earth Observatory Network, Nelson Mandela Metropolitan University, Port Elizabeth, South Africa; University of Pretoria, SOUTH AFRICA

## Abstract

This study aims to investigate macroinvertebrate assemblage structure and composition across the three major waterbody types (temporary rivers, depression wetlands and semi-permanent dams) of the Eastern Cape Karoo, and to identify important environmental and spatial correlates of macroinvertebrate assemblage composition in the region. A total of 33 waterbodies (9 dams, 13 depression wetlands and 11 rivers) were sampled. Altogether, 91 taxa were recorded in November 2014 and 82 in April 2015. Twenty-seven taxa were common to all three waterbody types (across both sampling occasions), with 17 of these observed in November and 19 in April. The ANOSIM tests revealed significant differences in assemblage composition between the depression wetlands and rivers for both sampling occasions, but dams did not differ from the other waterbody types. SIMPER analyses indicated that the notonectid *Anisops varia* and the corixid *Micronecta scutellaris* were abundant across all three waterbody types during both sampling occasions. The mayfly *Cloeon africanum and* the damselfly *Pseudagrion* sp. were abundant in river habitats during both sampling occasions, while the gastropod mollusc *Bulinus tropicus* and the copepod *Lovenula falcifera* best characterised depression wetlands on both occasions. Non-metric multidimensional scaling ordination highlighted a clear separation of assemblages between November and April, while distance-based Redundancy Analysis revealed that conductivity, altitude, turbidity and pH were the most important variables explaining the variation in macroinvertebrate assemblage patterns. These results provide baseline information which is important for future biological monitoring of impacts associated with hydraulic fracturing activities and climatic changes in the region.

## Introduction

Sustainable utilisation of freshwater systems requires knowledge of the spatial distribution of different water body types, the variability of their physico-chemistry across the landscape and the net contribution of each to catchment biodiversity [1]. Differences in local environmental conditions (e.g. habitat structural complexity and physico-chemistry) and the varying influence of biotic interactions result in distinct aquatic habitat types supporting different faunal assemblages [2–6]. Regional processes also influence species distributions, for example through colonization-extinction dynamics or dispersal limitation [7–9]. Inland waters are becoming increasingly threatened by anthropogenic activities such as water pollution, flow modification, destruction or degradation of habitat, invasion by exotic species, non-point impacts associated with land-use changes in catchments [10], and more recently, large-scale intensive hydraulic fracturing for shale gas extraction [11]. Despite their apparent vulnerability, there is little published information on the general ecology and biodiversity of small non-perennial freshwater systems characterising semi-arid regions, particularly in southern Africa [12–14].

Proposed hydraulic fracturing for shale gas in the interior Karoo region of South Africa has recently triggered much debate over its potential environmental effects, with impacts on water resources raising the greatest concern [15]. The region is also currently experiencing increases in mean annual temperatures [16], which are expected to prolong dry periods, potentially altering the hydrology and biodiversity of sensitive freshwater ecosystems. The Great Karoo Basin (hereafter ‘Karoo’) covers around 300 000 km^2^ of South Africa’s landmass and represents approximately 100 million years of sedimentation [17]. The interior semi-arid Karoo region is characterised by several freshwater systems such as non-perennial rivers, temporary depression wetlands and semi-permanent to permanent dams [18]. The intermittent rivers have a variable hydrologic cycle where phases of flow, no-flow and desiccation occur somewhat unpredictably, given the high patchiness of precipitation in time and space [19, 20]. This type of river is common in the Karoo region, and indeed in dryland areas worldwide [19, 21, 22]. Depression wetlands (sometimes locally referred to as ‘pans’) are common aquatic habitat features in South Africa, particularly in flat areas such as the interior plateau and lowland coastal plains [14, 23–26]. A depression wetland is defined by the recently developed South African wetland classification system of Ollis et al. [18] as “an inland aquatic ecosystem with closed (or near-closed) elevation contours, which increases in depth from the perimeter to a central area of greatest depth, and within which water typically accumulates”. During the last century, dams have become a characteristic aquatic feature of Karoo agricultural landscapes. Dams are artificially constructed reservoirs that in the Karoo may be isolated systems (fed by borehole water), or as is often the case, they are constructed across a river channel or unchannelled valley bottom wetland (*sensu* Ollis et al. [18]. The important role of dams as biodiversity refugia in semi-arid areas is now becoming recognised, given that they increase the availability of permanent or semi-permanent aquatic habitat and thus area of occupancy for many invertebrate species that rely on a more permanent aquatic medium for survival [27, 28].

Aquatic invertebrates are the most ubiquitous and diverse component of small non-perennial waterbodies of semi-arid and arid regions and have important potential for use in biological assessment of human impacts in such regions [24, 29–31]. This study has arisen largely from the need to establish baseline information on the diversity and distribution of Karoo wetland invertebrate assemblages, in order to facilitate future biological monitoring of impacts associated with hydraulic fracturing activities and climatic changes. The sub-region of the Greater Karoo Basin that falls within the Eastern Cape Province, the proposed epicentre of shale gas extraction activities in South Africa [32, 33], forms the focus of this investigation. We aim to: (i) assess aquatic macroinvertebrate assemblage composition and diversity across the three major waterbody types (temporary rivers, depression wetlands and semi-permanent dams) of the Eastern Cape Karoo; and (ii) identify prevailing environmental and spatial correlates of macroinvertebrate assemblage composition in the region. In order to sample the maximum diversity and abundance of aquatic macroinvertebrates, the study is focussed on periods where peak rainfall coincides with the warmest temperatures in the region.

## Materials and methods

### Ethics statement

Permission for fieldwork and scientific collection of macroinvertebrates in the Eastern Cape Karoo region earmarked for shale gas exploration was granted by the Eastern Cape Department of Economic Affairs, Environmental Affairs and Tourism (Cacadu Region) and access to privately owned land in the province of the Eastern Cape was granted by AGRI Eastern Cape. Permission to work in the Mountain Zebra National Park was granted by South African National Parks. The research involved the capture and handling of invertebrates, as approved in terms of animal care and use procedures by the Nelson Mandela Metropolitan University Ethics Committee (ethics clearance reference number: A14-SCI-ZOO-010).

### Study area

**Fig 1:**
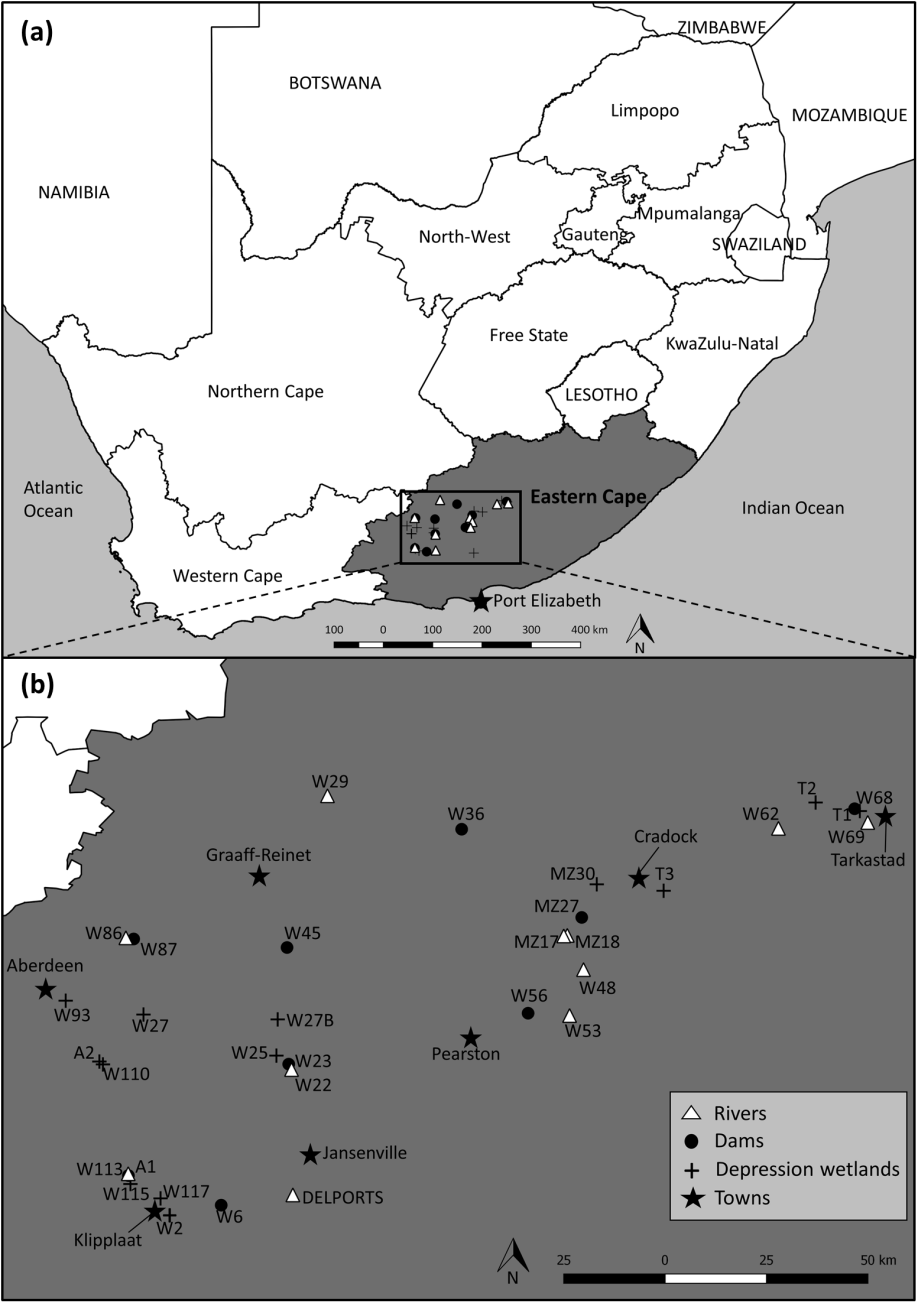
Location of sampling sites in the Eastern Cape Karoo region of South Africa. (a), and a zoomed -in perspective (b) of the 33 sites within the study area. Adapted from Mabidi et al. [39] under a CC BY license, with permission from Pensoft Publishers Ltd, original copyright 2016.

This survey investigates 33 waterbodies, including 9 dams, 13 depression wetlands and 11 rivers. All sites are located in an area of approximately 2 000 km^2^, bounded by the towns of Aberdeen in the west and Tarkastad in the east, and covering the bulk of the Eastern Cape Karoo region earmarked for potential shale gas exploration (Fig 1; see S1 Table for site coordinates). The area lies between 384 and 1450 m elevation in the Nama Karoo biome, which is dominated by low-shrub vegetation (< 1m tall) intermixed with grasses, succulents, geophytes and annual forbs. Tree occurrence in the biome is limited to habitats with unique hydropedological characteristics, for instance along drainage lines and rocky outcrops [34, 35]. The region is covered by shallow, weakly developed lime-rich soils underlain by sediments of the Karoo Supergroup (mostly glacial, shale and sandstone deposits). Air temperatures are highly variable, both diurnally and inter-seasonally, with extremes ranging from −5°C in winter to 43°C in summer [35, 36]. The bulk of the rainfall in the region occurs in summer, peaking between December and April [36], although sporadic rainfall events may occur throughout the year. Mean annual precipitation ranges from 70 mm in the west and south-west, to approximately 400 mm in the north-east of the study area, with a coefficient of variation of 30−60% [37, 38]. Low-intensity rangeland agriculture is the dominant land-use activity in the region, accompanied by sparse extents of irrigation agriculture and mining [35].

Field sampling was repeated on two occasions at the same sites in November 2014 (spring) and April 2015 (autumn). These sampling occasions were chosen so as to coincide with warm temperatures (i.e. excluding winter) and with maximum water availability to ensure that sites were visited during periods of peak invertebrate activity and biodiversity. During the April survey, one of the depression wetlands had dried up and one of the river sites could not be accessed. Thus, 31 of the original 33 sites were re-sampled in April.

### Physico-chemistry

Various measurements in the water column and bottom substrate were taken at each waterbody, in order to provide a physico-chemical context for the environments inhabited by the macroinvertebrate assemblages. Dissolved oxygen concentration, salinity, electrical conductivity, pH, turbidity and temperature were measured at three sampling locations in the limnetic zone of each site using an YSI 6600-V2 multiprobe system. An average of the three sampling values was used for further analyses. Three sediment core samples (3 cm^3^ each) were taken per site for the determination of benthic microalgal biomass. The cores were immediately placed in 90% acetone and stored in the dark below 4°C in the field, and subsequently stored at −19°C in the field laboratory within 8 hrs of collection. A 2 L integrated sample of surface water was collected from the water column across the full spatial extent of each site for laboratory analysis of nutrients, suspended solids and water column chlorophyll *a*. Water samples for measurement of chlorophyll *a* and suspended solids were immediately stored in the dark below 4°C and analysed within 24 h in the laboratory. A 300 ml water subsample was taken for the determination of total suspended solids (TSS) and particulate organic matter (POM), and was analysed using the American Public Health Association (APHA) method 2540, as described in Eaton et al. [40]. Another 300 ml subsample was filtered through a 0.7 μm glass-fibre filter (Whatman GF/F), the filtrand and filtrate of which were stored in the dark at −19°C for further nutrient analyses (all samples for nutrients were fully analysed in the laboratory within 14 days of sampling). The filtrand was analysed for ammonium (NH_4_^+^ −N) and soluble reactive phosphorus (orthophosphate, PO_4_^3+^ −P) using standard spectrophotometric methods as described by Parsons et al. [41]. Total oxidised nitrogen was measured using the reduced copper cadmium method as described by Bate and Heelas [42]. Chlorophyll *a* and phaeopigments were extracted from the filtrate using 90% acetone and measured using a Turner Designs fluorometer (model 10-AU) fitted with a narrow band non-acidification system [43], following the standard methods of Holm-Hansen and Riemann [44].

### Habitat cover

Habitat cover at each site was estimated by recording the cover of each of the three major habitat structural types encountered during this study. These were complex vegetation (generally submerged), simple vegetation (generally emergent) and benthic unvegetated (no vegetation). Complex vegetation habitat typically consisted of facultative aquatic macrophytes, such as *Stuckenia pectinata* and *Isolepis* spp. However, various other vegetation species also formed a complex submerged habitat, including flooded semi-aquatic and terrestrial grasses. Simply-structured vegetation habitat was typically rooted and emerging from the water surface and consisted of reeded forms (predominantly *Phragmites australis* and *Typha capensis*) and round-stemmed rush species (predominantly *Juncus* spp.). The cover of each of the habitat categories was recorded on an ordinal scale for each site as: 0 (not present); 1 (sparse); 2 (moderate); 3 (extensive) and 4 (complete cover). The areal cover of floating macroalgal mats was also recorded at each site on a scale of 0–4 as for vegetation cover.

### Hydro-morphometry, geology and land use

The surface area of lentic waterbodies was calculated using a handheld GPS device (Garmin eTrex Vista HCx, ± 3 m point accuracy) by walking the perimeter of the waterbody. For small depression wetlands, a tape measure was used to measure the dimensions of the wetland to improve the accuracy of surface area calculations. For larger dams, surface area was computed using satellite imagery in Google Earth Pro (Google Inc., 2016). The maximum depth of each waterbody was measured using a depth stick, operated from an inflatable boat in the case of large dams. Given that rivers are longitudinal features, the total river surface area in the catchment was not estimated and instead the surface area for a selected 50 m reach of river, where sampling activities took place, was measured. This measure provided a surface area proxy for comparisons among the river sites sampled in this study and thus does not represent an absolute measurement of surface area for comparison with other studies. At each river, we first selected an accessible 50 m reach where all sampling (including physico-chemistry and macroinvertebrates) was conducted. We then measured mean river width for the selected reach and multiplied this by the length of the reach (i.e. 50 m) to obtain a relative estimate of surface area that enabled comparisons with the surface areas of other river sites. Mean width at each site was calculated by measuring river width at five transects across the river, each at 10 m intervals along the selected 50 m reach. The underlying geology of each sampling site and the surrounding terrestrial vegetation type was ascertained by overlaying sites onto the South African lithological map (1:250 000 scale, Council for Geoscience map numbers 3324, 3224, 3126 and 3124) and the vegetation map of South Africa (1:1 000 000 scale vegetation map available from the South African National Biodiversity Institute, [45] in QGIS 2.2.0 Valmiera (QGIS, 2009). An estimate of the degree of land-use impact within 500 m of each waterbody was visually assessed using four ordinal categories: 0 (none); 1 (low); 2 (moderate); and 3 (high).

### Macroinvertebrates

All sites were divided into three size categories by approximate trisection of the range of surface area measures across all sites, resulting in small (< 499 m^2^), medium (500–1000 m^2^) and large (>1000 m^2^) sites. Macroinvertebrates were sampled semi-quantitatively with a D-frame sweep net (1 mm mesh size, 250 mm mouth diameter). Comparable sampling effort across all sites was ensured by means of a timed collection effort that was standardised according to the three size categories; small sites were vigorously swept for three minutes, medium-sized sites for six minutes and large sites for twelve minutes. Collected macroinvertebrates were preserved in 10% formalin on site and transported as such to the laboratory. Macroinvertebrates (defined as taxa >1 mm in size and visible to the naked eye) were identified and enumerated using a sub-sampling procedure. First, the whole sample was scanned for five minutes in a tray and large rare macroinvertebrate taxa (defined as taxa with large easily visible specimens represented by < 10 individuals per sample) were picked out in accordance with the recommendations of Vinson and Hawkins [46] and King and Richardson [47]. The sample was then emptied into a rectangular tray divided into a grid of 96 equal-sized square cells, which were randomly sub-sampled until 300 organisms had been picked out [47]. Sub-sampling stopped when 300 individuals had been counted, after first completing the cell in which the 300th individual was counted. The sub-sampling procedure of collecting the 300 individuals is widely used in biodiversity studies and, when complemented with a whole sample scan for rare species, generally produces reliable results [47]. According to Barbour and Gerritsen [48], if fixed-count sub-sampling for samples is conducted in an unbiased manner using a random selection method, the resulting information on richness and relative abundance is comparable among samples and produces stable values for metrics, when compared to larger or total counts [49]. Macroinvertebrate abundances were extrapolated to whole sample estimates by multiplying by the total number of cells, in order to standardise final abundances. Samples with < 300 individuals were completely picked. Organisms were identified to the lowest possible taxonomic level using the series of ‘Guides to the Freshwater Invertebrates of Southern Africa’ [50–58] as well as in consultation with relevant taxonomic experts. Most identifications were to genus or species level, with the exception of the chironomid larvae (Diptera), which could only be identified to subfamily level.

### Data analysis

For each of the three waterbody types, we calculated: 1) mean taxon richness (α-diversity), as the mean number of taxa collected per site; 2) regional taxon richness (γ-diversity), as the total number of taxa collected for each of the three waterbody types; and 3) taxon turnover (β-diversity), as regional richness divided by mean local richness (α-diversity). We perfomed individual-based rarefaction on the data to account for sampling biases, because of the different number of waterbody types involved in the investigation [59]. Differences in local taxon richness among the three waterbody types for both raw and rarefied data were examined for the November and April datasets separately using a non-parametric ANOVA (Kruskal-Wallis test), followed by *post hoc* multiple comparison tests where significant differences occurred. Mann-Whitney U tests were used to test for differences in taxon richness between the two sampling periods for each depression wetland and river, given that these waterbodies did not contain equal numbers of sites between the two sampling events. Inter-sampling differences in macroinvertebrate richness for dams were tested using Wilcoxon Matched-Pair tests, given the equal number of sites sampled on each trip for this waterbody type. The non-parametric asymptotic estimator first order Jacknife (Jacknife 1) was used to estimate total regional diversity (γ-diversity). This method uses information on the frequency of rare species in a sample to estimate the number of undetected species in a sample [60]. All univariate tests were performed using Statistica version 12 software for Windows (StatSoft Inc. 2015). Rarefaction analysis and estimation of species richness was carried out using PRIMER v6 software [61] and the rarefaction curves were created using the vegan package in the statistical software R version 3.3.3 (The R Foundation for Statistical Computing Platform 2017).

Analyses of macroinvertebrate assemblage differences among waterbody types were performed on the resemblance matrix based on the Bray-Curtis similarity coefficient [61], calculated on log-transformed macroinvertebrate abundance data. The resemblance matrix was then used to perform a non-metric multidimensional scaling (MDS) ordination to illustrate differences in macroinvertebrate assemblage composition between waterbody types and sampling events. The ‘Analysis of Similarities’ (ANOSIM) procedure [61] was then carried out to test for differences between macroinvertebrate assemblages among the three waterbody types, analysing the November and April datasets separately. ANOSIM calculates a test statistic R, which ranges from −1 to +1. Values approaching |1| indicate good separation of the groups and values approaching 0 indicate weak separation [61]. We performed the ‘Similarity Percentages’ (SIMPER) procedure [61], to quantify taxa contributing to the average Bray-Curtis similarity/dissimilarity among sites within each waterbody type or between all pair of sites among the three waterbody types. We then tested for differences in assemblage composition between the two sampling events (November vs April) using nonparametric permutational MANOVA (PERMANOVA, [62]. In order to account for assemblage differences due to waterbody type, we employed a two-way factorial design, which incorporated the factors ‘waterbody type’ (three levels–dams, depression wetlands and rivers) and ‘sampling event’ (two levels, November 2014 and April 2015). Residuals were permuted under a reduced model (9999 permutations). To perform the factorial MANOVA, the abundance data were first converted to a Bray-Curtis dissimilarity matrix.

In order to investigate environmental and spatial correlates of macroinvertebrate assemblages in Karoo waterbodies, we related the transformed (presence/absence) compositional abundance data (November and April datasets analysed separately) to the various measured environmental and spatial variables using distance-based Redundancy Analysis (dbRDA, [63, 64]. dbRDA is a non–parametric multivariate regression procedure based on any given dissimilarity measure, in this case the Bray–Curtis coefficient. Environmental predictor variables were log_10_ transformed where appropriate, in order to achieve normality. We used a step-wise regression procedure with an Akaike Information Criterion, corrected for small sample size (AICc), as the selection criterion to derive the most parsimonious subset of predictor variables associated with the macroinvertebrate assemblages [65]. P values for dbRDA models were tested by 9999 permutations of residuals under the reduced model. MDS, ANOSIM and SIMPER analyses were performed with PRIMER v6 software [61, 66]. Permutational MANOVA and dbRDA were performed using the PERMANOVA+ add-on package to PRIMER v6 [67]. The *a priori* significance level for all statistical tests in this study was set at α = 0.05.

## Results

### New species and distribution records

Four new/undescribed macroinvertebrate species were collected during this study and are currently being described by various taxonomic experts: one naucorid hemipteran in the genus *Laccocoris* (P. Reavell, Cape Town, pers. comm.); one helophorid beetle in the genus *Helophorus* (D. Bilton, Plymouth University, pers. comm.); and two mayflies in the genus *Caenis* (H. Barber-James, Albany Museum, pers. comm.). In terms of the large branchiopod fauna, new distribution records for the laevicaudatan *Lynceus truncatus* (Barnard, 1924) and the two spinicaudatans *Streptocephalus spinicaudatus* (Hamer & Appleton, 1993) and *Streptocephalus indistinctus* (Barnard, 1924) represent a substantial expansion of the previously known ranges for these species [39]. Tarkastad is now the westernmost record for *S*. *spinicaudatus*, while Jansenville now constitutes the southernmost record for *S*. *indistinctus*.

### Taxonomic richness across waterbody types

**Fig 2:**
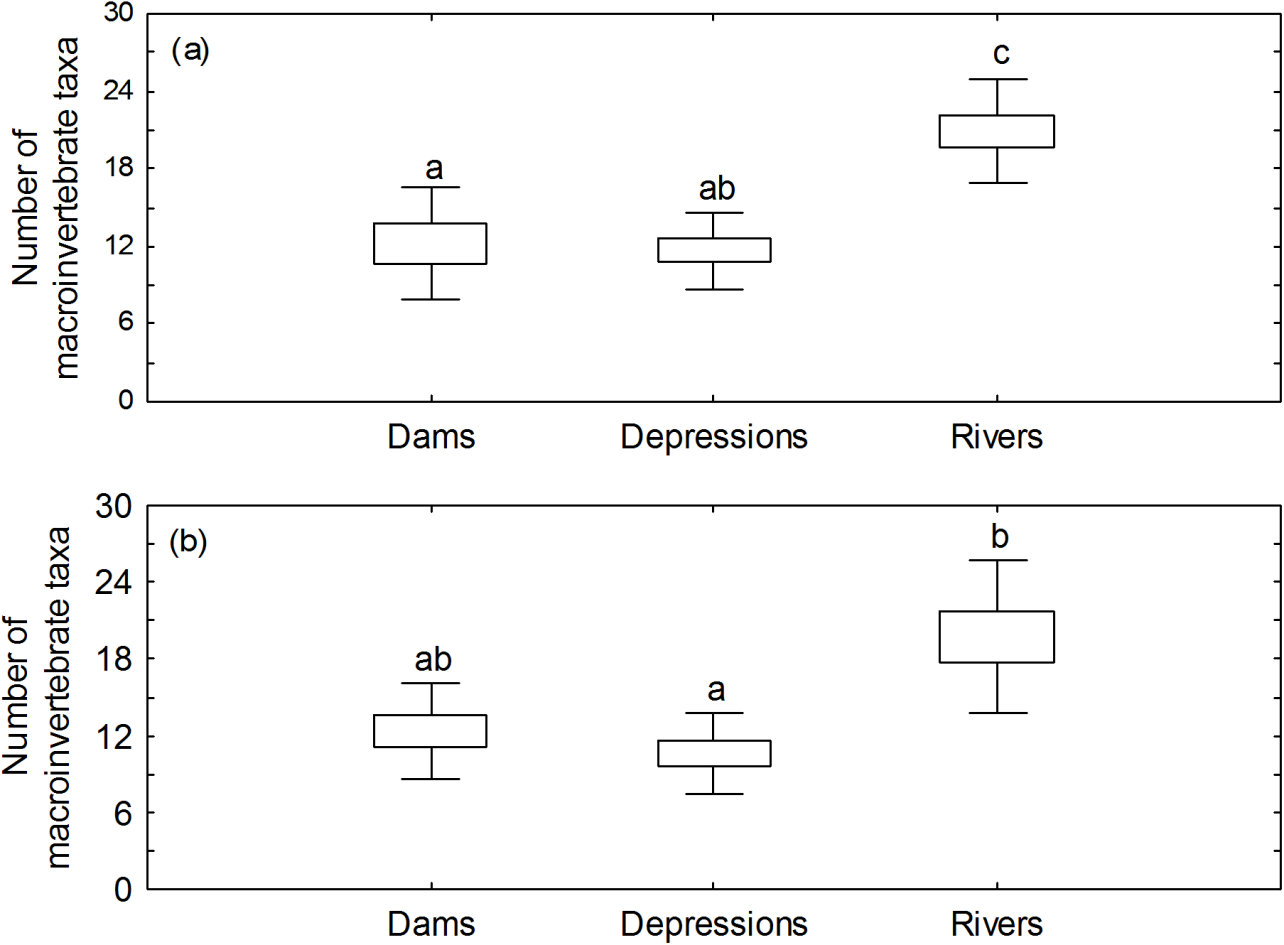
**Boxplots presenting summaries of the distribution of local taxon richness (α-diversity) in each waterbody type for (a) November 2014, and (b) April 2015.** Different letters denote significant differences at *p* < 0.05; error bars show the standard error of the mean and Depressions = depression wetlands.

There was a consistent pattern in local taxon richness (α-diversity) during both sampling occasions, with rivers supporting the highest richness and depression wetlands the lowest (Table 1). Even though slight shifts in mean richness were produced with rarefied data, the Kruskal-Wallis ANOVA tests produced results similar to those observed for raw richness (see S2 Table for full outputs of the analyses). There was a significant difference among the three water body types in November (Kruskal-Wallis H_2,33_ = 15.89, p = 0.0004). *Post hoc* multiple comparisons (Fig 2) indicated a significant difference in richness between dams and rivers (p = 0.004) and between depression wetlands and rivers (*p* = 0.001). A significant difference in richness among water body types (Kruskal-Wallis H_2,31_ = 13.01, *p* = 0.002) was also reported for April, with *post hoc* comparisons (Fig 2) indicating that on this occasion a significant difference existed only between depression wetlands and rivers (p = 0.001). There was however no significant difference in taxon richness for each of the waterbody types between the two sampling occasions (Mann-Whitney U-tests: U = 63.50, p = 0.43 for depression wetlands and U = 46.00, p = 0.53 for rivers; Wilcoxon Matched-Pair test: Z = 0.14, p = 0.889 for dams). Results were similar for both raw and rarefacted data (see S3–S5 Tables for full outputs of the analyses).

**Table 1:** Macroinvertebrate local taxon richness (α–diversity) mean and standard deviation for the three waterbody types in November 2014 and April 2015.

		Dams	Depressions	Rivers
**November 2014**	Raw data	12.22 ± 4.52	11.69 ± 3.15	20.91 ± 4.30
	Rarefacted data	7.86 ± 2.41	6.84 ± 2.06	11.93 ±3.11
**April 2015**	Raw data	12.33 ± 3.87	10.58 ± 3.32	19.7 ± 6.27
	Rarefacted data	7.70 ± 2.68	6.79 ± 1.26	11.41 ± 3.76

Depressions = depression wetlands

**Fig 3:**
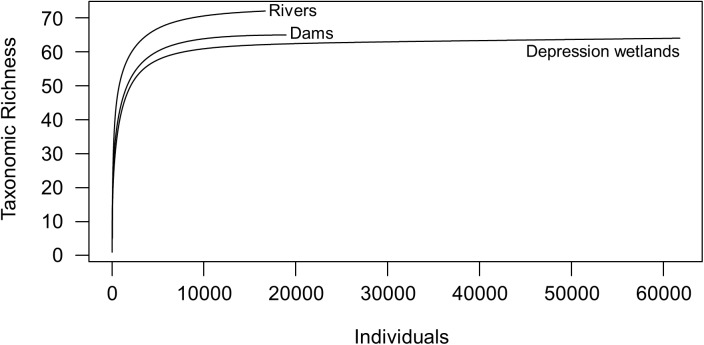
Individual-based rarefaction curves for macroinvertebrate taxon richness of the three waterbody types.

The same trend was obtained with regional diversity (γ-diversity), with rivers once again supporting the highest richness and depression wetlands the least (data from both sampling periods combined, Table 2). The rarefaction curves showed that an asymptote was reached only in depression wetlands, thus macroinvertebrate taxa remain to be discovered with increased sampling effort in dams and rivers (Fig 3). However, the predicted taxa richness by the Jacknife 1 richness estimator (Table 2) showed a different pattern to the observed data, with dams having the highest predicted taxa richness and depression wetlands the least. Despite depression wetlands being taxon poor at both local and regional diversity level, they had the highest taxon turnover while rivers had the least.

**Table 2:** Macroinvertebrate regional taxon richness (γ–diversity) and taxon turnover (β-diversity) for the three waterbody types.

		Dams	Depression wetlands	Rivers
Raw data	Regional taxon richness	65	64	72
	Taxon turnover	5.29	5.73	3.54
Jacknife 1 estimator	Regional taxon richness	92	82	83

Data combined for both November 2014 and April 2015. Predicted taxon richness by first order Jacknife estimator is also included.

### Assemblage composition

Taxa from 54 families were recorded across the two sampling occasions (see S6 Table). Dams contained 34 families, depression wetlands 29 and rivers 33 in November, while in April dams had 31 families and depression wetlands and rivers both had 33 families. Altogether, 91 taxa were recorded across the 33 waterbodies sampled in November 2014 and 82 in April 2015 (S6 Table). The predaceous beetle family Dytiscidae (Adephaga) was the richest group, with nine genera, followed by Baetidae (Ephemeroptera) with eight, Corixidae (Hemiptera) and Hydrophilidae (Polyphaga) with five each, Notonectidae (Hemiptera) and Streptocephalidae (Anostraca) with four each, and Caenidae (Ephemeroptera), Glossiphoniidae (Hirudinea), Gyrinidae (Adephaga) and Planorbidae (Gastropoda) with three each. Rivers supported the most exclusive taxa (28) followed by depression wetlands (17) and dams (7), (data from both sampling periods combined, S6 Table).

Depression wetlands supported the highest total macroinvertebrate abundance during both sampling occasions, while rivers had the lowest (S7 Table). Streptocephalidae (Anostraca) were the most numerous taxon in depression wetlands, while Culicidae (Diptera) were the most numerous in rivers. This trend held across both sampling occasions, with total abundances roughly doubling in April in each of these two waterbody types. However, in dams the most numerous taxa changed between seasons, with Notonectidae dominating total macroinvertebrate abundance in November and Branchipodidae (Anostraca) in April (S7 Table).

**Fig 4:**
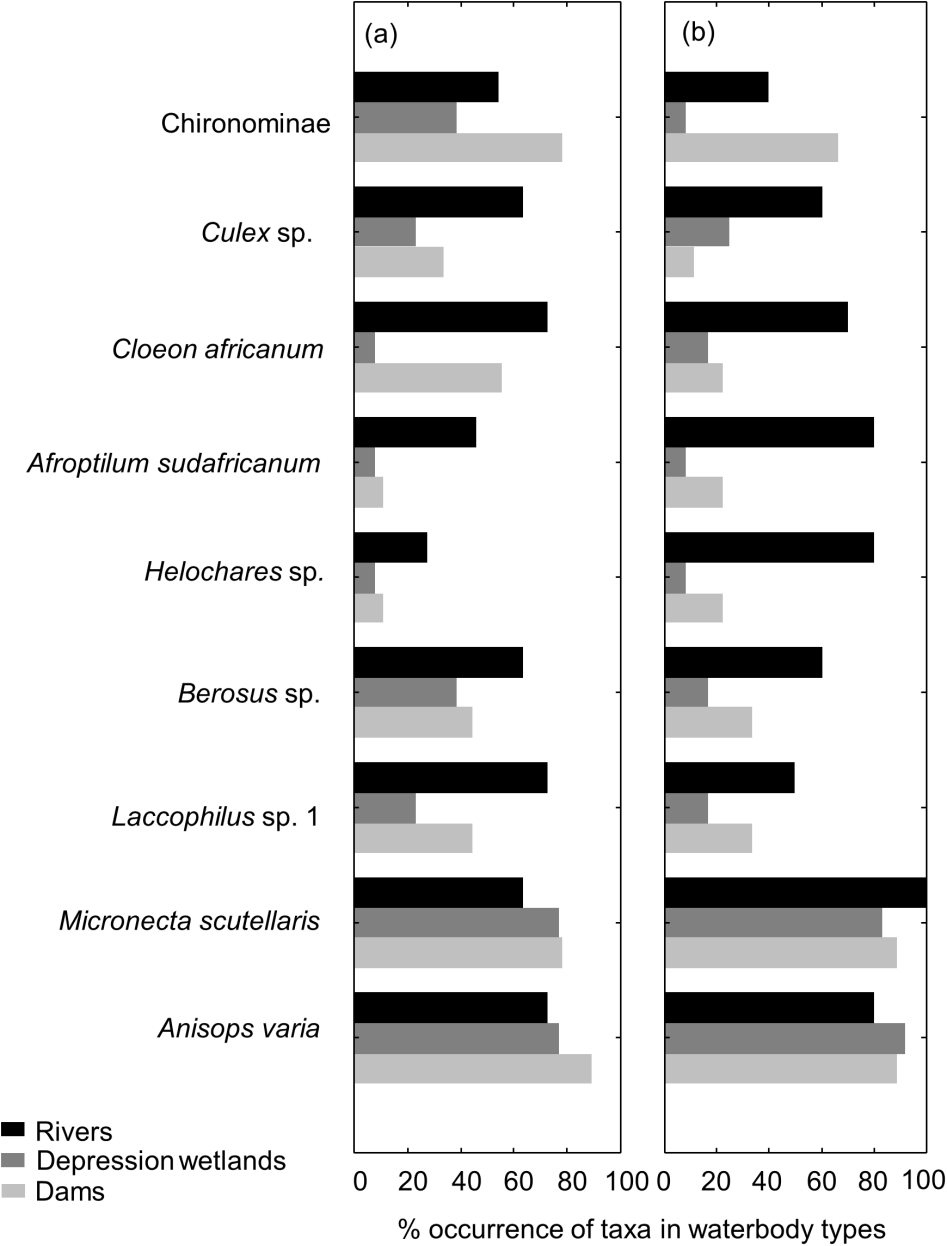
Proportion of occurrence of common macroinvertebrate taxa in each of the three waterbody types during (a) November 2014 and (b) April 2015.

Twenty-seven taxa were common to all three waterbody types (across both sampling occasions), with 17 of these being observed in November and 19 in April (Table 3). Nine of these 27 taxa were sampled on both the November and April collection trips. However, these nine taxa did not occur in all sites within each waterbody type (Fig 4). The notonectid hemipteran *Anisops varia* (Puton, 1899) and the corixid *Micronecta scutellaris* (Stål, 1858) occurred in more than 60% of individual sites for all three waterbody types during both seasons (Fig 4). The baetid mayfly *Afroptilum sudafricanum* (Lestage, 1924) was widespread in river sites, occurring in 46% of the sites in November and 80% in April, while it was found in less than 30% of depression wetlands and dams during both sampling occasions. Another baetid, *Cloeon africanum* (Esben-Petersen, 1913), as well as the hydrophilid beetle *Berosus* sp., the dytiscid beetle *Laccophilus* sp. 1 and the mosquito larvae of *Culex* sp. were all widespread in river sites only, where they occurred in more than 50% of the sites. Another hydrophilid, *Helochares* sp., was widespread in river sites (80% of the sites) in April only. Even though chironomid larvae of the sub-family Chironominae occurred in all three waterbody types, this taxon was only widespread in dams, occurring in more than 50% of dam sites during both sampling occasions.

**Table 3:** Macroinvertebrate taxa present in all three waterbody types during the two sampling occasions.

Taxa	November 2014	April 2015
*Placobdelloides multistriata* (Johansson, 1909)		+
*Lumbriculus variegatus* (Müller,1774)		+
*Bulinus tropicus* (Krauss, 1848)	+	
*Afroptilum sudafricanum* (Lestage, 1924)	+	+
*Cheleocloeon excisum* (Barnard, 1932)		+
*Cloeon africanum* (Esben-Petersen, 1913),	+	+
*Pseudocloeon latum* (Agnew, 1961)		+
*Mesocnemis* sp.	+	
*Pseudagrion* sp.	+	
*Anisops sardea* (Herrich-Schäffer, 1849)		+
*Anisops varia* (Fieber, 1851)	+	+
*Appasus capensis* (Mayr, 1843)		+
*Gerris swakopensis* (Stål, 1858)		+
*Micronecta citharistia* (Hutchinson, 1929)	+	
*Micronecta scutellaris* (Stål 1858)	+	+
*Plea pullula* (Stål, 1855)		+
*Sigara pectoralis* (Fieber, 1851)	+	
*Sigara wahlbergi* (Lundblad 1928)	+	
*Berosus* sp.	+	+
*Helochares* sp.	+	+
*Herophydrus inquinatus* (Boheman, 1848)		+
*Hydroglyphus lineolatus* (Boheman, 1848)	+	
*Nebrioporus vagrans* (Omer-Cooper, 1953)	+	
*Laccophilus* sp.1	+	+
*Laccocoris* sp.		+
Chironominae	+	+
*Culex* sp.	+	+

**Fig 5:**
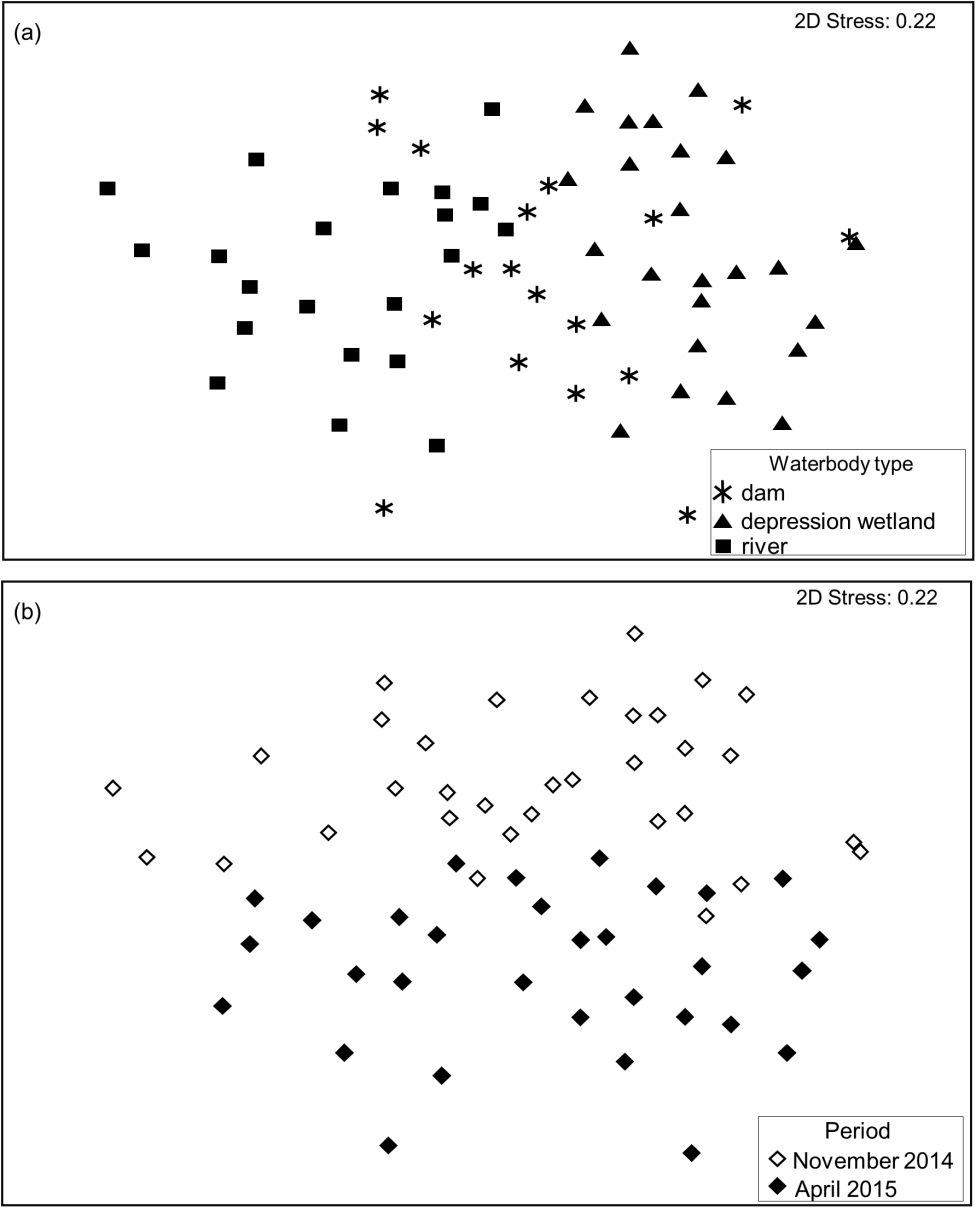
MDS ordination plot of macroinvertebrate assemblage composition based on Bray-Curtis similarity among sites. Plots depict (a) the three waterbody types, and (b) the two sampling occasions (November 2014 versus April 2015).

The ANOSIM tests showed significant differences in assemblage composition between the depression wetlands and rivers, during both November (R = 0.59, P = 0.001) and April (R = 0.84, P = 0.001), but dams did not differ from the other waterbody types (see S8 Table for full output of analyses). The lack of assemblage distinction between dams and other waterbody types is reflected by the partial overlap of dam sites with both depression wetlands and rivers on the MDS ordination (Fig 5). Results from SIMPER analyses are shown in Tables 4 and 5. The average Bray-Curtis similarity between all pairs of sites in dams was 31.62 in November and 30.24 in April, and was made up mainly of contributions from dysticid larvae and the corixid *Micronecta scutellaris* respectively. In depression wetlands, the average Bray-Curtis similarity was 33.25 in November and 33.10 in April, and was made up mainly of contributions from the corixid *Sigara pectoralis* and the notonectid *Anisops varia* respectively. *Enithares* nymphs and *Micronecta scutellaris* made up the main contributions to the average Bray-Curtis similarity of 33.58 in November and 33.50 in April in rivers respectively (Table 4). The average Bray-Curtis dissimilarities between all pairs of sites in dams and depression wetlands was 71.50 in November and 72.92 in April, and was made up mainly of contributions from *Lovenula falcifera* and *Streptocephalus* juveniles respectively. The average Bray-Curtis dissimilarity between dams and rivers was 72.96 in November and 74.87 in April and was made up mainly of contributions from *Lovenula falcifera* and *Streptocephalus* juveniles respectively. However, between depression wetlands and rivers, *Sigara pectoralis* and *Bulinus tropicus* contributed the most to the average Bray-Curtis dissimilarity of 80.96 in November and 83.58 in April (Table 5).

**Table 4:** SIMPER results listing the macroinvertebrate taxa that contributed to the average Bray-Curtis similarity among sites within each waterbody type.

Taxon	Dams		Depressions		Rivers	
	Nov 2014	April 2015	Nov 2014	April 2015	Nov 2014	April 2015
*Anisops varia*	10.86	16.20	10.12	18.38	6.73	9.01
*Micronecta scutellaris*	9.57	20.67	9.27	12.57	4.73	12.29
Dytiscid larvae	11.16			9.63	4.09	
Chironominae	9.64					
*Sigara* nymphs	11.11					
*Micronecta* nymphs		13.33				4.20
*Sigara pectoralis*			19.77		3.94	
*Bulinus tropicus*			11.84	16.91		
*Enithares* nymphs					7.64	
*Cloeon africanum*					7.30	9.74
*Pseudagrion* sp.					6.62	4.59
*Culex* sp.					3.99	
*Laccophilus* sp.1					3.65	
*Gerris swakopensis*					3.36	
*Afroptilum sudafricanum*						7.77
*Trithemis* sp.						4.36

Taxa are listed in terms of their percentage contribution to the Bray-Curtis similarity of each waterbody type. The given taxa contributed ~50% to the cumulative similarity for each waterbody type (November and April analysed separately). Depressions = depression wetlands.

**Table 5:** SIMPER results listing the macroinvertebrate taxa that contributed to the average Bray-Curtis dissimilarity between all pair of sites among the three waterbody types.

	Dams and Depressions		Dams and Rivers		Depressions and Rivers	
Macroinvertebrate taxa	November	April	November	April	November	April
*Lovenula falcifera*	4.53	3.54	2.50		3.40	2.70
*Bulinus tropicus*	4.05	2.78	2.31	3.08	3.56	3.92
*Anisops* nymphs	3.96	2.37	3.49	2.37	2.65	
*Enithares* nymphs	3.62		2.79		2.90	
*Sigara pectoralis*	3.62	2.34	2.86		3.63	2.16
Chironominae	3.59	2.42	3.40	2.79	1.89	1.77
*Cyzicus australis*	3.50	3.96			2.92	3.44
Dytiscid larvae	3.30	2.73	3.13		1.97	2.51
*Sigara* nymphs	3.14		2.68		2.64	
*Micronecta scutellaris*	3.04	2.77	3.09	2.71	2.40	2.01
*Culex* sp.	2.72		2.90	2.40	3.00	2.23
*Anisops varia*	2.64	2.76	2.45	2.98	2.19	2.41
Hydrophilid larvae	2.26				1.88	
*Sigara meridionalis*	2.17		2.08	1.78	1.60	
*Cloeon africanum*	2.12		2.55	3.70	2.69	3,26
*Pseudagrion* sp.	2.06	2.13	2.52	2.63	2.40	1.94
*Laccophilus* sp.1			2.16			
*Caenis subota*			2.06		1.64	
*Caenis* sp. 2			2.01		1.68	
*Trithemis* sp.			1.99	2.06		1.84
*Sigara wahlbergi*			1.98		1.75	
*Chlorolestes* sp.			1.93			
*Micronecta* nymphs		3.46		3.11	1.74	2.20
*Baetis harrisoni*					1.67	
*Streptocephalus* juveniles		4.18				3.34
*Branchipodopsis* juveniles		3.39				2.14
*Triops granarius*		3.08				2.36
*Leptestheria rubidgei*		2.45				
*Eocyzicus obliquus*		2.27				1.82
*Streptocephalus ovamboensis*		2.20				1.87
*Baetid* nymphs		2.14		2.10		
*Afroptilum sudafricanum*				2.84		2.58
*Pseudocloeon latum*				2.45		2.07
*Berosus* sp.				2.05		
*Cheleocloeon excisum*				2.03		1.75
*Helochares* sp.				1.82		
*Nychia limpida*				1.77		
*Cloeon* sp.2				1.77		
*Plea pullula*				1.71		
*Herophydrus inquinatus*				1.67		
*Lacoccoris* sp.				1.67		

Taxa are listed in terms of their percentage contribution to the average Bray-Curtis dissimilarity. Given are taxa with a cumulative contribution of ~50% of the total similarity/dissimilarity (November and April analysed separately). Depressions = depression wetlands.

The MDS ordination (Fig 5) showed a clear separation of assemblages between November and April. This separation was confirmed by the permutational MANOVA results (Table 6), which reported significant effects for both the ‘sampling event’ (F = 5.71, P = 0.0001) and ‘waterbody type’ (F = 7.29, P = 0.0001) factors, with the non-significant interaction term (F = 0.84, P = 0.7457) indicating that temporal differences were consistent across waterbody types and *vice versa*.

**Table 6:** Non-parametric permutational MANOVA results for the two-way factorial model examining the effect of waterbody type and season (sampling period) on macroinvertebrate assemblage composition of Eastern Cape Karoo waterbodies.

Both seasons combined Source	df	SS	MS	F	P
Waterbody type	2	33971	16986	7.29	**0.0001**
Season	1	13314	13314	5.71	**0.0001**
Waterbody type × Season	2	3936	1968	0.84	0.7457
Residual	58	135120	2330		
Total	63	186230			

Bold values indicate significant P values at α = 0.05.

### Environmental correlates of assemblage composition

**Fig 6:**
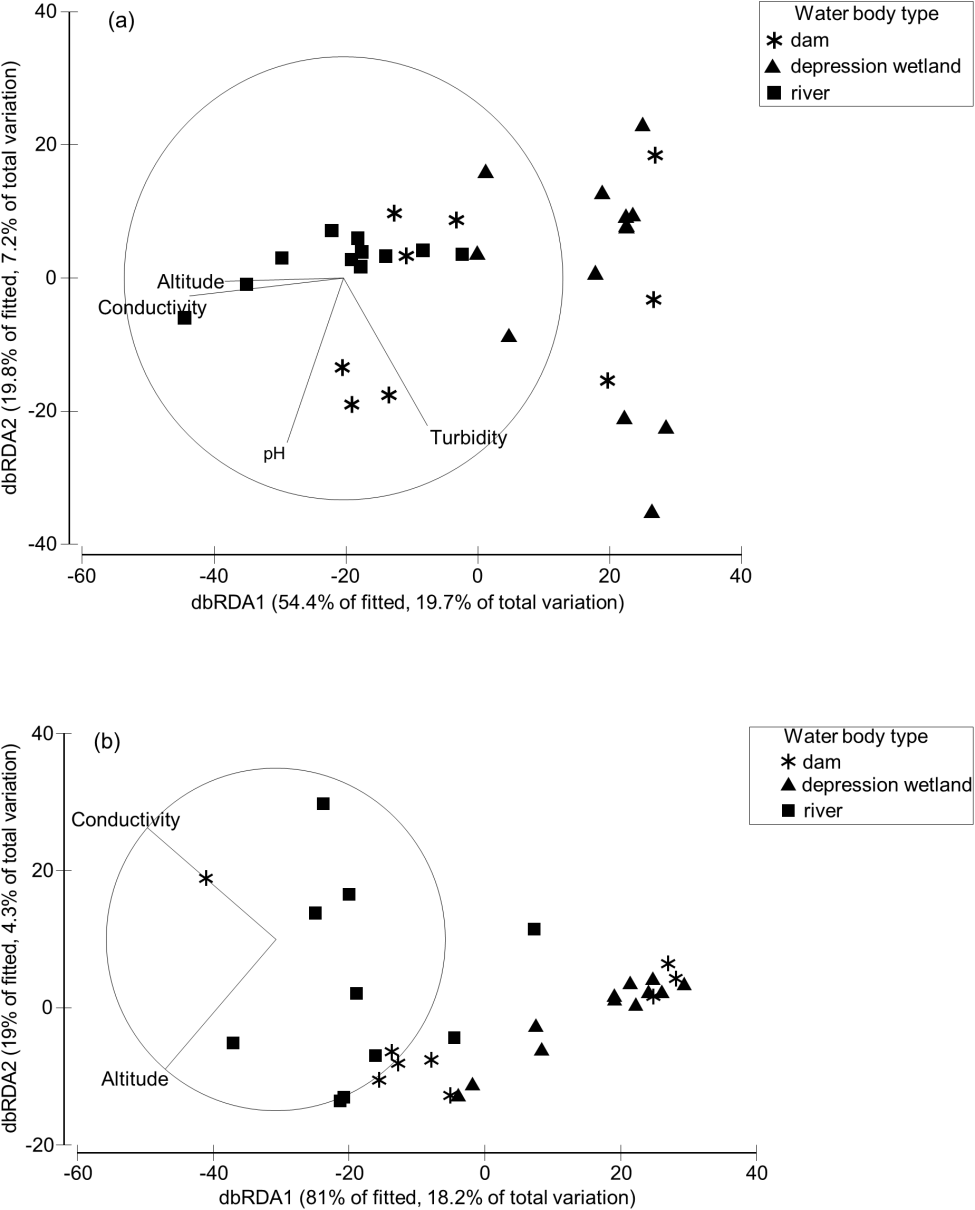
**dbRDA ordination plots (AICc selection criterion) of macroinvertebrate assemblage composition among sites (Bray–Curtis similarity) constrained by the environmental variables, November (a) and April (b).** Explained variation in the fitted model and total explained variation are indicated for each axis.

Conductivity, altitude, turbidity and pH were selected by the AICc criterion as significant correlates of assemblage composition in both November and April, with turbidity and pH only selected in November (Fig 6). The direction of the environmental vectors in both occasions indicates that turbidity influenced macroinvertebrate assemblage composition in depression wetlands, pH in dams, while conductivity and altitude influenced assemblages in rivers. However, all the vectors showed a weak relationship with the waterbody type gradient, particularly in April. The variables selected by the AICc criterion for the November dataset explained 36.28% of the cumulative variation in the macroinvertebrate assemblage composition (Table 7), while for April the selected variables explained 22.52% of the cumulative variation.

**Table 7:** Non-parametric multivariate regression results (dbRDA, AICc selection criteria) for environmental variables that best explained variation in macroinvertebrate assemblages in the Eastern Cape Karoo.

Period	Variable	AICc	F	*P*	%Var	Res df
November 2014	Conductivity	254.74	4.43	0.0001	12.50	31
	Altitude	252.78	4.27	0.0001	10.89	30
	Turbidity	252.43	2.72	0.0024	6.56	29
	pH	252.09	2.78	0.0005	6.33	28
April 2015	Conductivity	242.17	3.55	0.0007	10.92	29
	Altitude	240.31	4.19	0.0001	11.60	28

%Var = the percentage of variation that is explained by each respective predictor variable in each model; Res. df = residual degrees of freedom for each model; BU habitat = benthic unvegetated habitat.

## Discussion

The findings presented here provide the first account of the composition and ecology of macroinvertebrate assemblages in surface waterbodies of the Eastern Cape Karoo region of South Africa. The current survey offers initial insight into the composition of macroinvertebrate assemblages that characterise the Karoo waterbodies and some of the environmental determinants that appear to play a role in structuring these assemblages. During both the November 2014 and April 2015 sampling campaigns, macroinvertebrate assemblages were dominated in terms of abundance by large branchiopods (especially Streptocephalidae) in depression wetlands and insects (especially Culicidae) in rivers. In dams, insects (Notonectidae) dominated abundance in November 2014 and large branchiopods (Branchipodidae) in April 2015. Two hemipterans, the corixid *Micronecta scutellaris* and the notonectid *Anisops varia*, were recorded in all three waterbody types and were widespread among sites in each waterbody type. These taxa were widespread, but not necessarily locally abundant. This is in contrast to the commonly observed pattern of distribution for many organisms, where spatial distribution is positively correlated with average abundance [68–70].

The current study indicates that river sites had the highest local taxon richness (α-diversity), while depression wetlands had the least taxa. This could likely be attributed to most of the taxa in rivers being cyclic colonisers, with juvenile life stages that cannot withstand prolonged desiccation. Although many of the Karoo rivers investigated in this study do cease to flow during drought conditions, most appear to retain isolated pools of water during droughts (pers. obs.), which likely facilitates the persistence of desiccation-intolerant taxa. Therefore, river sites provide habitats with an increased length of the wet phase, which is the most stable environment for such taxa [71]. Depression wetlands are often characterised by a short hydroperiod, which limits colonisation and the establishment of species-rich stable communities, as relatively few species can tolerate the physico-chemical fluctuations associated with rapidly changing water levels [72, 73].

Regional taxon richness (γ-diversity) was also highest in rivers in comparison with the other two waterbody types. Similar findings were reported by Uys [20] for temporary rivers in the Eastern Cape and by Williams [21] for non-perennial rivers in Australia. The latter reported that relative to similar non-perennial river systems elsewhere in the world, these rivers are often characterized by a higher species diversity. They attributed this partly to the heterogeneity of surface water conditions over time. Because of the highly variable and unpredictable rainfall and flow rates, rivers present a suite of different hydraulic and substratum conditions that provide a large variety of niches for a diverse fauna [20]. Thus, in the present study, rivers continuously presented a heterogeneous habitat to which a variety of fauna may have been attracted at different times, and this possibly accounts for the high taxon diversity observed here. However, this is in contrast to findings by Williams et al. [1], who found an unusually high regional macroinvertebrate taxon richness (γ- diversity) in ponds, relative to the other waterbody types in southern England. They pointed out that the pattern could be driven by landscape factors, such as physico-chemical heterogeneity and pond connectivity to smaller catchments as factors that could be responsible for maintaining the ponds’ rich regional diversity [1]. However, the Eastern Cape Karoo is a semi-arid region where most of the rivers are seasonally intermittent and are considered unstable systems, in comparison with the more perennial rivers of temperate zones [20]. Even though increasing diversity is known to correspond with habitat stability and duration [74, 75], this is not the case in this region. Dams present the only relatively stable systems in the region, but were the least diverse in comparison with the other two waterbody types, making it difficult to predict diversity patterns. Even though all the waterbodies studied were reported to be consistently alkaline [76], dams had slightly higher alkalinity, which could explain the low macroinvertebrate diversity in these systems. Thus dams in the region may be important only as biodiversity refugia during drought [27, 28], increasing the availability of permanent or semi-permanent aquatic habitat for many invertebrate species that rely on a more permanent aquatic medium for survival.

Results from this study indicate that, despite depression wetlands having low mean local and regional richness, they have the highest taxon turnover, while rivers had the lowest. Macroinvertebrate assemblages in temporary depression wetlands of semi-arid regions are subjected not only to seasonal changes, but also to many environmental fluctuations on a shorter time scale, such as pronounced diurnal fluctuation in temperature and pH, as well as physico-chemical changes associated with drawdown of water levels, often on a timescale of weeks rather than months [73, 77]. This often leads to ongoing changes in the structure of macroinvertebrate assemblages in these systems [77, 78]. The depression wetlands investigated in this study were set apart from the other two waterbody types in the SIMPER analysis by the presence of the snail *Bulinus tropicus* (Krauss, 1848), which is a passive disperser. These results are in agreement with findings reported by other researchers [79–81]. Unlike active dispersers, which migrate between habitats depending on the wet phase [21], passive dispersers are exposed to continuous desiccation events. This moulds the life history traits of passive dispersers, thus making them more permanent inhabitants of temporary aquatic systems [79, 82]. It is also important to note that Dytiscidae and Corixidae juvenile forms were largely confined to dams and rivers. This could be because early life stages are unlikely to survive the dry phase within depression wetlands, which are short-lived systems [83].

All three waterbody types supported taxa that were unique to each, with rivers supporting the most unique taxa and dams the least. The distribution of macroinvertebrate taxa among the three waterbody types was not unexpected. Only the rivers for instance contained the mayfly *Adenophlebia sylvatica* (Crass, 1947) (Leptophlebiidae) the dragonflies *Aeshna* sp. (Aeshnidae) and *Paragomphus* sp. (Gomphidae), the blackfly *Simulium* sp. (Simuliidae), the whirligig beetles *Aulonogyrus alternatus* (Régimbart, 1892) and *Orectogyrus polli* (Régimbart, 1884) (Gyrinidae), which are all species typical of flowing, clear and cold waters [84]. Large branchiopods (crustacean class Branchiopoda) such as fairy shrimp (Anostraca), clam shrimp (Laevicaudata, Spinicaudata,) and tadpole shrimp (Notostraca), as well as the water scavenger beetle *Helophorus* sp. (Helophoridae) were confined to dams and depression wetlands. Large branchiopods are known as flagship inhabitants of temporary habitats [85], while the water scavenger beetle is known to reside in temporary water habitats [1].

Other than waterbody type, which appears to have an overarching influence on the types of macroinvertebrates in Karoo waterbodies, assemblage variation was best explained by conductivity, habitat cover (benthic unvegetated habitat being most influential) and altitude (Table 7). Of these factors, conductivity explained the most variation in macroinvertebrate composition, although even this was fairly low at 12.50% in November and 10.92% in April. The importance of conductivity was not surprising given that rivers had significantly higher conductivity levels than the other two waterbody types [76] and conductivity is a known determinant of macroinvertebrate assemblages elsewhere [86, 87]. The low richness and distinct macroinvertebrate assemblage composition of depression wetlands in the Karoo appears to be associated with the high turbidity (Fig 6) that characterizes these waterbodies [76]. The importance of water transparency in influencing the structure of invertebrate assemblages has been highlighted by several authors [88–91]. Depression wetlands were also characterised by benthic unvegetated habitat which explain the turbid nature of these systems. Macrophytes reduce current velocities both within and adjacent to the vegetation, resulting in increased sedimentation and reduced turbidity [92]. Macrophytes are also used by macroinvertebrates as refugia against predation and offer a substrate for living on [93], thus sites without macrophytes can be expected to support distinct assemblages. Particularly, small temporary depression wetlands generally have far fewer predators than larger more permanent systems [94], and despite the harsh environment in these wetlands, certain unique taxa exploit the opportunity to inhabit relatively predator-free environments (e.g. large branchiopods). pH was an important variable explaining the variation in macroinvertebrate assemblages in November (Table 7). The direction of the environmental vector indicates that pH influenced macroinvertebrate assemblage composition in dams (Fig 6). Several studies have also highlighted the importance of pH in structuring aquatic invertebrates [95–97]. All three waterbody types spanned a range of altitudes, although depression wetlands were more common in flat low-lying areas near the towns of Aberdeen, Klipplaat and Jansenville, while flowing rivers were generally more common in higher altitude areas. Altitude is a well-known determinant of aquatic macroinvertebrate assemblage composition [98–100] and the significant influence of this factor in large-scale studies covering a broad altitudinal range is not uncommon [101].

Macroinvertebrates are excellent candidates as indicators of the environmental impacts that may be associated with fracturing activities, given their use as biological indictors of other human impacts in various freshwater environments [102, 103]. The impending hydraulic fracturing activities in the Karoo region may pose potential impacts on surface freshwater systems. There is currently little consensus on just how much impact these activities are likely to have on surface waters, given the highly recent and rapid development of fracturing initiatives elsewhere (e.g. North America) and thus the lack of baseline monitoring of aquatic ecological impacts to date [104]. There is some evidence that the release of hypersaline flowback and disposal of untreated wastewater from shale gas operations can impact the inorganic quality of surface water [105–107]. According to Nielsen and Brock [105], increases in surface water salinity pose the greatest threat to the biodiversity of freshwater environments, given that increasing salinity in freshwater systems often leads to reductions in biodiversity. Large branchiopods, which are flagship inhabitants of temporary depression wetlands, and were also found in dams in this study, are particularly at risk. These crustaceans rely on banks of resting eggs as a buffer against environmental variability [85]. Although generally resilient to harsh environmental conditions, large branchiopods are sensitive to increases in salinity. Under increasing salinity conditions these organisms are unlikely to hatch optimally and survive until reproduction [87, 108]. This may cause depletion of the resting egg bank and eventually extirpation of local populations, thereby affecting the whole wetland community through cascading effects on other faunal and floral components [87].

Sedimentation, which can result from habitat clearance for shale gas development, can also increase turbidity in the surface freshwater systems, particularly in the Eastern Cape Karoo where soils are often fine and clayey and thus runoff is likely to cause heavy sedimentation of nearby waterbodies. Surface freshwater bodies in the Karoo region are turbid [76], and turbidity was one of the environmental factors that explained a significant variation in the macroinvertebrate assemblages in the present study. The detrimental effect of fine suspended sediments on abundance and diversity of macroinvertebrates has been widely reported [91, 109–111]. It was not unexpected that river sites contained much of the larval stages of most insects in comparison with the other two waterbody types. Larvae of almost all species of dragonflies and damselflies (Odonata) are dependent on freshwater habitats and are susceptible to changes in water flow, turbidity or loss of aquatic vegetation [84]. Thus, possible changes in salinity and turbidity constitute important threats to macroinvertebrate biodiversity in the region.

### Conclusions

The results obtained in the first survey of the surface waters of the Eastern Cape Karoo indicate that the three waterbody types in the region support diverse macroinvertebrate assemblages, each contributing taxa not found in other habitats. However, as evidenced by the collection of four undescribed species, more information is required on the taxonomy, distribution and status of the Karoo macroinvertebrates fauna. The bulk of macroinvertebrate species collected during the present study are widespread taxa which are not rare, threatened or endemic. Rivers had the most macroinvertebrate taxa, while dams had the least and contained macroinvertebrate taxa that exhibited elements of either depression wetlands or rivers. Dams in the region have been found to exhibit physico-chemical characteristics intermediate between rivers and depression wetlands [76], thus it was not unexpected that they also contained taxa which were similar to either. Dams also appear to be an important habitat for invertebrates, particularly for water-scavenger beetles (family Helophoridae) and large branchiopods. Other authors have highlighted the importance of dams in terms of their contribution to regional biodiversity in semi-arid regions [27, 112]. These waterbodies provide important semi-permanent refugia for aquatic macroinvertebrates and thus, although they are artificial features, they should be considered in future conservation initiatives for the region. Differences in macroinvertebrate richness, abundance and distribution patterns among sites were only weakly influenced by local and regional environmental factors.

To enable the data presented in this study to contribute to a powerful assessment tool of future environmental impacts on surface water environments of the Eastern Cape Karoo, further studies are required that more closely investigate how macroinvertebrate assemblages vary in space and time in relation to the environmental variables in this relatively unstudied region. It must be emphasized that future long-term monitoring should build directly on this initial dataset by sampling these sites, or a subset thereof, over an extensive period (ideally 10 years or more), accounting for seasonal variation within each year of monitoring. Long-term monitoring studies will enhance our understanding of factors that structure macroinvertebrate assemblages in the region and can lead to better-informed assessments of hydraulic fracturing impacts or those related to climate change. This is particularly necessary for depression wetlands because the assemblages are unstable as indicated by the high taxon turnover between years observed in the current study. Furthermore, depression wetlands are the most at risk to impacts typically associated with hydraulic fracturing pollution because of their relatively small volume and shallow depth. In particular, salinisation can be anticipated to have detrimental effects on the macroinvertebrate inhabitants of these depression wetlands. To effectively monitor these effects, more studies are needed, with focus on factors structuring macroinvertebrate assemblages in depression wetlands, particularly large branchiopods because these might serve as indicators of pollution.

## Supporting information

S1 Table Location of sampled waterbodies (latitude and longitude readings at the centre point of each site in decimal degrees). Depressions = depression wetlands.
(DOCX)

S2 Table Kruskal-Wallis outputs (both raw and rarefacted data) of comparisons of local macroinvertebrate taxa (α- diversity) among the three waterbody types for November 2014 and April 2015. (DOCX)

S3 Table Mann-Whitney tests outputs (both raw and rarefacted data) of comparisons of local macroinvertebrate taxa (α- diversity) for depression wetlands between November 2014 and April 2015. (DOCX)

S4 Table Mann-Whitney tests outputs (both raw and rarefacted data) of comparisons of local macroinvertebrate taxa (α- diversity) of rivers between November 2014 and April 2015. (DOCX)

S5 Table Full statistical output for the results of the Wilcoxon Matched-Pair tests (both raw and rarefacted data) for differences in local macroinvertebrate taxa (α- diversity) between the November 2014 and April 2015. (DOCX)

S6 Table Macroinvertebrate taxa collected in the three waterbody types in the Eastern Cape Karoo region earmarked for shale gas exploration during the November 2014 and April 2015. a = present only in April; n = present only in November; an = present during both periods; 0 = absent during both periods; Exclusive = exclusive to indicated waterbody type; Dep = depression wetlands; Riv = rivers.
(DOCX)

S7 Table Macroinvertebrate taxa (family level) relative abundances for each waterbody type during the two sampling occasions. Bold values indicate families with the highest number of individuals per each water body type.
(DOCX)

S8 Table Statistical output for the results of the ANOSIM tests for differences between macroinvertebrate assemblages among the three waterbody types, analysing the November 2014 and April 2015 datasets separately. Depression = depression wetland.
(DOCX)

S1 Text Permission to publish a modified version of the map (Fig 1) under PLOS’ CC BY 4.0 license. (PDF)
