# Supplementary material for: Distribution and diversity of aquatic macroinvertebrate assemblages in a semi-arid region earmarked for shale gas exploration (Eastern Cape Karoo, South Africa)
Source: PLoS One. 2017 Jun 2;12(6):e0178559. doi: 10.1371/journal.pone.0178559 (PMC5456075; doi:10.1371/journal.pone.0178559)
Supplement: S1 Table — Depressions = depression wetlands. (DOCX) [file pone.0178559.s001.docx]

**S1 Table. Location of sampled waterbodies (latitude and longitude readings at the centre point of each site in decimal degrees).**

| **Waterbody type** | Site code | Latitude | Longitude | Altitude (m.a.s.l) |
| --- | --- | --- | --- | --- |
| **Dams** | A1 | −32.921278 | 24.260071 | 674 |
|  | MZ27 | −32.237188 | 25.460087 | 1228 |
|  | T1 | −31.949062 | 26.18231 | 1279 |
|  | W6 | −32.999809 | 24.505765 | 537 |
|  | W23 | −32.626008 | 24.684467 | 582 |
|  | W36 | −32.003417 | 25.142233 | 1194 |
|  | W56 | −32.491048 | 25.318032 | 1175 |
|  | W45 | −32.316885 | 24.679974 | 1311 |
|  | W87 | −32.294236 | 24.274062 | 796 |
| **Depressions** | A2 | −32.619112 | 24.183249 | 731 |
|  | MZ30 | −32.149391 | 25.49982 | 1008 |
|  | T2 | −31.932568 | 26.079092 | 1217 |
|  | T3 | −32.166526 | 25.676791 | 930 |
|  | W2 | −33.027906 | 24.345178 | 616 |
|  | W25 | −32.603381 | 24.651856 | 598 |
|  | W27 | −32.494572 | 24.299996 | 661 |
|  | W27B | −32.507438 | 24.655214 | 654 |
|  | W68 | −31.955212 | 26.196083 | 1275 |
|  | W93 | −32.457843 | 24.094083 | 738 |
|  | W110 | −32.626712 | 24.192674 | 725 |
|  | W115 | −32.943724 | 24.265472 | 669 |
|  | W117 | −32.981841 | 24.345178 | 641 |
| **Rivers** | Delports | −32.972683 | 24.695368 | 385 |
|  | MZ17 | −32.286219 | 25.412386 | 1434 |
|  | MZ18 | −32.284753 | 25.42182 | 1417 |
|  | W22 | −32.640334 | 24.691929 | 578 |
|  | W29 | −31.914737 | 24.787045 | 1357 |
|  | W48 | −32.375479 | 25.46477 | 1204 |
|  | W53 | −32.497168 | 25.427859 | 1028 |
|  | W62 | −32.001047 | 25.980677 | 1066 |
|  | W69 | −31.985479 | 26.217119 | 1228 |
|  | W86 | −32.291238 | 24.25339 | 788 |
|  | W113 | −32.916513 | 24.259611 | 680 |

Depressions = depression wetlands.
