# Supplementary material for: Distribution and diversity of aquatic macroinvertebrate assemblages in a semi-arid region earmarked for shale gas exploration (Eastern Cape Karoo, South Africa)
Source: PLoS One. 2017 Jun 2;12(6):e0178559. doi: 10.1371/journal.pone.0178559 (PMC5456075; doi:10.1371/journal.pone.0178559)
Supplement: S2 Table — (DOCX) [file pone.0178559.s002.docx]

**S2 Table. Kruskal-Wallis outputs (both raw and rarefacted data) of comparisons of local macroinvertebrate taxa (α- diversity) among the three waterbody types for November 2014 and April 2015.**

|  |  | November 2014 |  |  |  | April 2015 |  |  |  |
| --- | --- | --- | --- | --- | --- | --- | --- | --- | --- |
|  | Variable | H_2,33_ | *P*-value | Post hoc *P*-value | Significantly different waterbody types | H_2,31_ | *P*-value | Post hoc *P*-value | Significantly different waterbody types |
| Raw data | α-diversity | 15.89 | 0.0004 | 0.0043 | Dams and Rivers | 13.01 | 0.0015 | 0.0012 | Depression wetlands and Rivers |
|  |  |  |  | 0.0008 | Depression wetlands and Rivers |  |  |  |  |
| Rarefacted data |  | 14.59 | 0.0007 | 0.0250 | Dams and Rivers | 10.16 | 0.0062 | 0.0062 | Depression wetlands and Rivers |
|  |  |  |  | 0.0006 | Depression wetlands and Rivers |  |  |  |  |
