# Supplementary material for: Distribution and diversity of aquatic macroinvertebrate assemblages in a semi-arid region earmarked for shale gas exploration (Eastern Cape Karoo, South Africa)
Source: PLoS One. 2017 Jun 2;12(6):e0178559. doi: 10.1371/journal.pone.0178559 (PMC5456075; doi:10.1371/journal.pone.0178559)
Supplement: S3 Table — (DOCX) [file pone.0178559.s003.docx]

**S3 Table. Mann-Whitney tests outputs (both raw and rarefacted data) of comparisons of local macroinvertebrate taxa (α- diversity) for depression wetlands between November 2014 and April 2015.**

| Variable | Rank Sum | Rank Sum | U | Z | p-level | Z adjusted | p-level | Valid N | Valid N | 2-sided exact p |
| --- | --- | --- | --- | --- | --- | --- | --- | --- | --- | --- |
| α-diversity | November | April |  |  |  |  |  | November | April |  |
| Raw data | 183.50 | 141.50 | 63.50 | 0.788696 | 0.430290 | 0.793440 | 0.427555 | 13 | 12 | 0.437102 |
| Rarefacted data | 168.00 | 157.00 | 77.00 | -0.054393 | 0.956622 | -0.054393 | 0.956622 | 13 | 12 | 0.978697 |
