# Supplementary material for: Distribution and diversity of aquatic macroinvertebrate assemblages in a semi-arid region earmarked for shale gas exploration (Eastern Cape Karoo, South Africa)
Source: PLoS One. 2017 Jun 2;12(6):e0178559. doi: 10.1371/journal.pone.0178559 (PMC5456075; doi:10.1371/journal.pone.0178559)
Supplement: S4 Table — (DOCX) [file pone.0178559.s004.docx]

**S4 Table. Mann-Whitney tests outputs (both raw and rarefacted data) of comparisons of local macroinvertebrate taxa (α- diversity) of rivers between November 2014 and April 2015.**

|  | Variable | Rank Sum | Rank Sum | U | Z | p-level | Z adjusted | p-level | Valid N | Valid N | 2-sided exact p |
| --- | --- | --- | --- | --- | --- | --- | --- | --- | --- | --- | --- |
|  | α-diversity | November | April |  |  |  |  |  | November | April |  |
| Raw data |  | 130.00 | 101.00 | 46.00 | 0.633761 | 0.526237 | 0.637707 | 0.523665 | 11 | 10 | 0.557264 |
| Rarefacted data |  | 126.00 | 105.00 | 50.00 | 0.352089 | 0.724771 | 0.352089 | 0.724771 | 11 | 10 | 0.756444 |
