# Supplementary material for: Distribution and diversity of aquatic macroinvertebrate assemblages in a semi-arid region earmarked for shale gas exploration (Eastern Cape Karoo, South Africa)
Source: PLoS One. 2017 Jun 2;12(6):e0178559. doi: 10.1371/journal.pone.0178559 (PMC5456075; doi:10.1371/journal.pone.0178559)
Supplement: S5 Table — (DOCX) [file pone.0178559.s005.docx]

**S5 Table. Full statistical output for the results of the Wilcoxon Matched-Pair tests (both raw and rarefacted data) for differences in local macroinvertebrate taxa (α- diversity) between the November 2014 and April 2015.**

| November and April α-diversity | Valid N | T | Z | p-level |
| --- | --- | --- | --- | --- |
| Raw data | 9 | 17.00 | 0.140028 | 0.888638 |
| Rarefacted data | 9 | 20.00 | 0.296174 | 0.767097 |
