# Supplementary material for: Distribution and diversity of aquatic macroinvertebrate assemblages in a semi-arid region earmarked for shale gas exploration (Eastern Cape Karoo, South Africa)
Source: PLoS One. 2017 Jun 2;12(6):e0178559. doi: 10.1371/journal.pone.0178559 (PMC5456075; doi:10.1371/journal.pone.0178559)
Supplement: S6 Table — a = present only in April; n = present only in November; an = present during both periods; 0 = absent during both periods; Exclusive = exclusive to indicated waterbody type; Dep = depression wetlands; Riv = rivers. (DOCX) [file pone.0178559.s006.docx]

**S6 Table.** **Macroinvertebrate taxa collected in the three waterbody types in the Eastern Cape** **Karoo region earmarked for shale gas exploration during the November 2014 and April 2015.**

| **Family** | **Taxon** | **Dams** | **Dep** | **Riv** | **Exclusive** |
| --- | --- | --- | --- | --- | --- |
| Hydrachnidae | *Hydrachna* sp. | 0 | an | 0 | Dep |
| Hygrobatidae | *Atractides* sp. | 0 | an | n | Dep |
| Lycosidae | *Pardosa* sp*.* | 0 | a | a |  |
| Streptocephalidae | *Streptocephalus cafer* Lovén, 1847 | 0 | an | 0 | Dep |
|  | *Streptocephalus indistinctus* Barnard, 1924 | a | an | 0 |  |
|  | *Streptocephalus ovamboensis* Barnard, 1924 | n | an | 0 | Dep |
|  | *Streptocephalus spinicaudatus* Hamer & Appleton, 1993 | 0 | n | 0 |  |
| Branchipodidae | *Branchipodopsis wolfi* Daday, 1910 | 0 | an | 0 | Dep |
| Lynceidae | *Lynceus truncatus* Barnard, 1924 | 0 | a | 0 | Dep |
| Cyzicidae | *Cyzicus australis* Loven, 1847 | an | an | 0 |  |
|  | *Eocyzicus obliquus* Sars, 1905 | a | a | 0 |  |
| Leptestheriidae | *Leptestheria inermis* Barnard, 1929 | n | 0 | 0 |  |
|  | *Leptestheria rubidgei* Baird, 1862 | a | a | 0 |  |
|  | *Leptestheria striatoconcha* Barnard, 1924 | 0 | a | 0 | Dep |
| Limnadiidae | *Eulimnadia* sp*.* | 0 | n | 0 |  |
| Triopsidae | *Triops granarius* Lucas, 1864 | an | an | 0 |  |
| Diaptomidae | *Lovenula falcifera Lovén*, 1845 | an | an | 0 |  |
| Daphniidae | *Daphnia carinata* King, 1852 | 0 | n | 0 | Dep |
|  | *Daphnia dolichocephala* Sars, 1895 | 0 | 0 | 0 |  |
|  | *Simocephalus serrulatus* Koch, 1841 | a | 0 | a |  |
| Curculionidae | *Neochetina* sp. | an | an | 0 |  |
| Dytiscidae | *Cybister tripunctatus africanus* Laporte, 1835 | 0 | n | 0 | Dep |
|  | *Eretes sticticus* Linnaeus, 1767 | n | n | 0 |  |
|  | *Herophydrus* sp. | a | a | an | Riv |
|  | *Hydaticus dregei* Aubé, 1838 | 0 | 0 | an | Riv |
|  | *Hydaticus servillanus* Aubé, 1838 | 0 | 0 | a | Riv |
|  | *Hydroglyphus lineolatus* Boheman, 1848 | n | n | n |  |
|  | *Laccophilus* sp.1 | an | an | an |  |
|  | *Laccophilus* sp.2 | a | 0 | an | Riv |
|  | *Nebrioporus vagrans* Omer-Cooper, 1953 | n | an | an |  |
|  | *Ranthus* sp. | a | an | 0 | Dep |
| Gyrinidae | *Aulonogyrus abdominalis* Aubé, 1838 | an | 0 | an |  |
| **Family**  **S6 Table continued Appendix 1** continued | **Taxon** | **Dams** | **Dep** | **Riv** | **Exclusive** |
|  | *Aulonogyrus alternatus* Régimbart, 1892 | 0 | 0 | an | Riv |
|  | *Orectogyrus polli* Régimbart, 1884 | 0 | 0 | an | Riv |
| Helophoridae | *Helophorus* sp*.* nov | n | an | 0 | Dep |
| Hydrophilidae | *Berosus punctulatus* Boheman, 1851 | n | 0 | 0 | Dams |
|  | *Berosus* sp*.* | an | an | an |  |
|  | *Helochares* sp*.* | an | an | an |  |
|  | *Hydrochara flavipalpis* Boheman, 1851 | n | 0 | n |  |
|  | *Paracymus pisanus* Balfour-Browne, 1954 | 0 | 0 | n | Riv |
| Spercheidae | *Spercheus cerisyi* Guérin-Méneville 1842 | 0 | an | an |  |
| Potamonautidae | *Potamonautes sidneyi* Rathbun, 1904 | 0 | 0 | an | Riv |
| Ceratopogonidae | *Bezzia* sp. | n | 0 | an |  |
| Chironomidae | Chironominae | an | an | an |  |
|  | Tanypodinae | an | 0 | 0 | Dams |
| Culicidae | *Anopheles* sp. | 0 | 0 | an | Riv |
|  | *Culex* sp. | an | an | an |  |
| Dixidae | *Dixa* sp. | an | 0 | n |  |
| Limoniidae | *Limonia tipulipes* Karsch, 1886 | a | 0 | a |  |
| Simuliidae | *Simulium* sp. | 0 | 0 | an | Riv |
| Tipulidae | *Tipula* sp. | an | 0 | an |  |
| Baetidae | *Afroptilum sudafricanum* Lestage, 1924 | an | a | an |  |
|  | *Baetis harrisoni* Barnard, 1932 | 0 | a | an |  |
|  | *Cheleocloeon excisum* Barnard, 1932 | a | a | an | Riv |
|  | *Cloeon africanum* Esben-Petersen, 1913 | an | a | an |  |
|  | *Cloeon* sp. 1 | a | n | an |  |
|  | *Cloeon* sp. 2 | an | 0 | a | Dams |
|  | *Pseudocloeon latum* Agnew, 1961 | a | a | an | Riv |
|  | *Pseudocloeon* sp. | 0 | 0 | an | Riv |
| Caenidae | *Caenis subota* Malzacher, 2012 | n | 0 | an | Riv |
|  | *Caenis* sp. 1 | 0 | 0 | an | Riv |
|  | *Caenis* sp. 2 | 0 | 0 | an | Riv |
| Leptophlebiidae | *Adenophlebia sylvatica* Crass, 1947 | 0 | 0 | an | Riv |
| Lymnaeidae | *Lymnaea columella* Say, 1817 | 0 | an | n | Dep |
| Physidae | *Physa acuta* Draparnaud, 1805 | 0 | a | an | Riv |
| Planorbidae | *Bulinus forskalii* Ehrenberg, 1831 | 0 | 0 | n | Riv |
|  | *Bulinus tropicus* Krauss, 1848 | an | an | an |  |
|  | *Burnupia* sp. | 0 | 0 | n | Riv |
| Belostomatidae | *Appasus capensis* Mayr, 1843 | a | a | an | Riv |
| **Family** | **Taxon** | **Dams** | **Dep** | **Riv** | **Exclusive** |
| Corixidae | *Micronecta citharistia* Hutchinson, 1929 | n | n | n |  |
|  | *Micronecta scutellaris* Stål, 1858 | an | an | an |  |
|  | *Sigara meridionalis* Wallengren, 1875 | an | an | 0 |  |
|  | *Sigara pectoralis* Fieber, 1851 | an | an | an |  |
|  | *Sigara wahlbergi* Lundblad, 1928 | an | n | n | Dams |
| Gerridae | *Gerris swakopensis* Stål, 1858 | a | an | an |  |
| Naucoridae | *Laccocoris* sp. | an | a | an |  |
| Nepidae | *Laccotrephes* sp. | a | an | 0 | Dep |
| Notonectidae | *Anisops sardea* Herrich-Schäffer, 1849 | a | an | an |  |
|  | *Anisops varia* Fieber, 1851 | an | an | an |  |
|  | *Enithares chinai* Jaczewski 1927 | 0 | an | an |  |
|  | *Nychia limpida* Stål, 1860 | an | 0 | an |  |
| Paraphyrynoveliidae | *Paraphyrynovelia* sp. | 0 | 0 | a | Riv |
| Pleidae | *Plea pullula* Stål, 1855 | an | a | an |  |
| Hydridae | *Hydra* sp. | 0 | 0 | n | Riv |
| Lumbriculidae | *Lumbriculus variegatus* Müller,1774 | a | a | a |  |
| Aeshnidae | *Aeshna* sp. | 0 | 0 | an | Riv |
|  | *Anax* sp. | n | 0 | an | Riv |
| Coenagrionidae | *Pseudagrion* sp. | an | 0 | an |  |
| Corduliidae | *Phyllomacromia* sp. | n | 0 | 0 | Dams |
| Gomphidae | *Crenigomphus* sp. | 0 | 0 | n |  |
|  | *Paragomphus* sp. | 0 | 0 | an | Riv |
| Lestidae | *Lestes* sp*.* | n | a | n | Dep |
| Libellulidae | *Trithemis* sp. | an | 0 | an |  |
| Platycnemididae | *Mesocnemis* sp*.* | n | n | n |  |
| Synlestidae | *Chlorolestes* sp*.* | n | 0 | an | Riv |
| Hirudinidae | *Hirudo michaelseni* Augener, 1936 | n | an | 0 | Dep |
| Glossiphoniidae | *Alboglossiphonia disjuncta* Moore, 1939 | 0 | an | 0 | Dep |
|  | *Marsupiobdella africana* Goddard & Malan, 1912 | 0 | n | 0 | Dep |
|  | *Placobdelloides multistriata* Johansson, 1909 | an | an | a |  |
|  | *Theromyzon cooperi* Harding, 1932 | a | 0 | 0 | Dams |
| Hydropsychidae | *Cheumatopsyche* sp*.* | a | 0 | an | Riv |
| Dugesiidae | *Dugesia* sp*.* | n | 0 | 0 | Dams |

a = present only in April; n = present only in November; an = present during both periods; 0 = absent during both periods; Exclusive = exclusive to indicated waterbody type; Dep =depression wetlands; Riv = rivers.

**S6 Table continued Appendix 1** continued
