# Supplementary material for: Distribution and diversity of aquatic macroinvertebrate assemblages in a semi-arid region earmarked for shale gas exploration (Eastern Cape Karoo, South Africa)
Source: PLoS One. 2017 Jun 2;12(6):e0178559. doi: 10.1371/journal.pone.0178559 (PMC5456075; doi:10.1371/journal.pone.0178559)
Supplement: S7 Table — Bold values indicate families with the highest number of individuals per each water body type. (DOCX) [file pone.0178559.s007.docx]

**S7 Table.** **Macroinvertebrate taxa (family level) relative abundances for each waterbody type during the two sampling occasions.**

|  | Dams |  | Depression wetlands |  | Rivers |  |
| --- | --- | --- | --- | --- | --- | --- |
| Family | November 2014 | April 2015 | November 2014 | April 2015 | November 2014 | April 2015 |
| Notonectidae | **4188** | 5396 | 6150 | 1860 | 610 | 649 |
| Corixidae | 2116 | 2421 | 6490 | 2186 | 660 | 587 |
| Pleidae | 396 | 86 | 0 | 31 | 9 | 14 |
| Nepidae | 0 | 10 | 20 | 2 | 0 | 0 |
| Naucoridae | 21 | 142 | 0 | 19 | 35 | 44 |
| Belastomatidae | 0 | 90 | 32 | 24 | 2 | 12 |
| Gerridae | 0 | 12 | 11 | 52 | 92 | 47 |
| Paraphyrynoveliidae | 0 | 0 | 0 | 0 | 0 | 6 |
| Platycnemididae | 5 | 0 | 69 | 0 | 5 | 0 |
| Synlestidae | 34 | 0 | 0 | 0 | 137 | 15 |
| Coenagrionidae | 128 | 839 | 112 | 0 | 226 | 142 |
| Lestidae | 271 | 0 | 0 | 51 | 5 | 0 |
| Gomphidae | 0 | 0 | 16 | 0 | 19 | 16 |
| Aeshnidae | 10 | 0 | 0 | 0 | 25 | 78 |
| Libellulidae | 342 | 23 | 0 | 0 | 25 | 136 |
| Corduliidae | 5 | 0 | 0 | 0 | 0 | 0 |
| Triopsidae | 1 | 221 | 368 | 1342 | 0 | 0 |
| Streptocephalidae | 926 | 2253 | **8903** | **21749** | 0 | 0 |
| Branchipodidae | 121 | **6067** | 1951 | 14716 | 0 | 0 |
| Cyzicidae | 10 | 48 | 1167 | 5543 | 0 | 0 |
| Limnadiidae | 0 | 0 | 11 | 0 | 0 | 0 |
| Leptestheriidae | 5 | 509 | 0 | 1522 | 0 | 0 |
| Lynceidae | 0 | 0 | 0 | 12 | 0 | 0 |
| Daphniidae | 0 | 3 | 1063 | 0 | 0 | 3 |
| Diaptomatidae | 2233 | 98 | 8815 | 3175 | 0 | 0 |
| Potamonautidae | 0 | 0 | 0 | 0 | 14 | 41 |
| Gyrinidae | 9 | 19 | 0 | 0 | 98 | 57 |
| Dytiscidae | 1206 | 119 | 447 | 464 | 293 | 174 |
| Hydrophilidae | 63 | 268 | 483 | 260 | 62 | 101 |
| Helophoridae | 17 | 0 | 145 | 45 | 0 | 0 |
| Curculionidae | 1 | 8 | 65 | 105 | 0 | 0 |
| Spercheidae | 177 | 9 | 137 | 122 | 39 | 33 |
| Baetidae | 199 | 582 | 373 | 411 | 1364 | 2227 |
| Leptophlebiidae | 0 | 0 | 0 | 0 | 208 | 213 |
| Caenidae | 30 | 0 | 0 | 0 | 853 | 181 |
| Planorbidae | 134 | 357 | 2730 | 1291 | 324 | 36 |
| Lymnaeidae | 0 | 0 | 13 | 32 | 17 | 0 |
| Physidae | 0 | 0 | 0 | 24 | 354 | 115 |
| Hydrachnidae | 0 | 0 | 26 | 18 | 0 | 0 |
| Hygrobatidae | 0 | 0 | 22 | 14 | 1 | 0 |
| Lycosidae | 0 | 0 | 0 | 19 | 0 | 7 |
| Ceratopogonidae | 5 | 0 | 0 | 0 | 23 | 2 |
|  | Dams |  | Depression wetlands |  | Rivers |  |
| Family | November 2014 | April 2015 | November 2014 | April 2015 | November 2014 | April 2015 |
| Culicidae | 63 | 61 | 5080 | 59 | **2043** | **6414** |
| Dixidae | 10 | 6 | 0 | 0 | 15 | 0 |
| Simuliidae | 0 | 0 | 0 | 0 | 36 | 15 |
| Chironomidae | 1522 | 367 | 260 | 13 | 119 | 383 |
| Tipulidae | 5 | 28 | 0 | 0 | 7 | 47 |
| Limoniidae | 0 | 7 | 0 | 0 | 0 | 2 |
| Lumbriculidae | 0 | 292 | 0 | 267 | 0 | 2 |
| Hydropsychidae | 0 | 7 | 0 | 0 | 1 | 2 |
| Hydridae | 0 | 0 | 0 | 0 | 4 | 0 |
| Dugesiidae | 5 | 0 | 0 | 0 | 0 | 0 |
| Glossiphoniidae | 5 | 12 | 670 | 33 | 0 | 3 |
| Hirudinidae | 2 | 0 | 5 | 12 | 0 | 0 |
| Total | 14266 | 20357 | 45637 | 55476 | 7724 | 11804 |

Bold values indicate families with the highest number of individuals per each water body type.

**S7 Table continued Appendix 1** continued
