# Supplementary material for: Distribution and diversity of aquatic macroinvertebrate assemblages in a semi-arid region earmarked for shale gas exploration (Eastern Cape Karoo, South Africa)
Source: PLoS One. 2017 Jun 2;12(6):e0178559. doi: 10.1371/journal.pone.0178559 (PMC5456075; doi:10.1371/journal.pone.0178559)
Supplement: S8 Table — Depression = depression wetland. (DOCX) [file pone.0178559.s008.docx]

**S8 Table.** **Statistical output for the results of the ANOSIM tests for differences between macroinvertebrate assemblages among the three waterbody types, analysing the November 2014 and April 2015 datasets separately.**

|  | November 2014 |  | April 2015 |  |
| --- | --- | --- | --- | --- |
| Pairwise Tests | R Statistic | Significance Level | R Statistic | Significance Level |
| Dam, Depression | 0.206 | 0.011 | 0.291 | 0.002 |
| Dam, River | 0.233 | 0.009 | 0.382 | 0.001 |
| Depression, River | 0.587 | 0.001 | 0.841 | 0.001 |

Depression = depression wetland.
