# Supplementary material for: Distribution and diversity of aquatic macroinvertebrate assemblages in a semi-arid region earmarked for shale gas exploration (Eastern Cape Karoo, South Africa)
Source: PLoS One. 2017 Jun 2;12(6):e0178559. doi: 10.1371/journal.pone.0178559 (PMC5456075; doi:10.1371/journal.pone.0178559)
Supplement: S1 Text — (PDF) [file pone.0178559.s009.pdf]

Pavel Stoev <projects@pensoft.net>
to me, ZooKeys

Pavel Stoev <projects@pensoft.net>

to: annah anusa <annahanusa@gmail.com>, ZooKeys <zookeys@pensoft.net>

date: Mon, Apr 3, 2017 at 10:22 AM

subject: Re: Request for permission to reproduce a map

mailed-by: pensoft.net

signed-by: pensoft.net

Dear Annah Mabidi,

Thank you for your inquiry.

You may re-use the map provided the original source is duly acknowledged.

Best wishes,

Pavel

--
Prof. Pavel Stoev
Editorial Director
Pensoft Publishers Ltd.
12, Prof. G. Zlatarski St., 1700 Sofia, Bulgaria
(+359-2) 8704281 (tel.); 8704282 (fax)
E-mail: projects@pensoft.net

annah anusa <annahanusa@gmail.com>
to ZooKeys

annahanusa <annahanusa@gmail.com>

to: ZooKeys <zookeys@pensoft.net>

date: Mon, Apr 3, 2017 at 8:48 AM

subject: Request for permission to reproduce a map

mailed-by: gmail.com

Dear Pensoft Publishers,

“I request permission for the open-access journal PLOS ONE to publish a map similar to the one published in Mabidi A, Bird MS, Perissinotto R, Rogers DC (2016) Ecology and distribution of large branchiopods (Crustacea, Branchiopoda, Anostraca, Notostraca, Laevicaudata, Spinicaudata) of the Eastern Cape Karoo, South Africa. ZooKeys 618: 15–38. doi: 10.3897/zookeys.618.9212 under the Creative Commons Attribution License (CCAL) CC BY 4.0 (http://creativecommons.org/licenses/by/4.0/). Please be aware that this license allows unrestricted use and distribution, even commercially, by third parties. Please reply and provide explicit written permission to publish the Figure 1. (Map) under a CC BY license.”

I look forward to hearing from you.

With thanks and regards,

Annah

Annah Mabidi
PhD student (DST/NRF Research Chair: Shallow Water Ecosystems)
C/o Department of Zoology
Nelson Mandela Metropolitan University
Summerstrand South Campus
P.O.Box 77000 Port Elizabeth 6031
South Africa
Cell:+27 76 710 3471
Email: s214371387@nmmu.ac.za
